# Drought Analysis Using Standardized Evapotranspiration and Aridity Index at Bilate Watershed: Sub-Basins of Ethiopian Rift Valley

**DOI:** 10.1155/2022/1181198

**Published:** 2022-02-14

**Authors:** Bereket Tesfaye Haile, Kassahun Ture Bekitie, Tadesse Terefe Zeleke, Desalegn Yayeh Ayal, Gudina Legese Feyisa, Fikiru Abiko Anose

**Affiliations:** ^1^Centre for Environmental Science, College of Natural and Computational Science, Ababa University, Addis, Ethiopia; ^2^Department of Ecobiology, College of Applied Sciences, Addis Ababa Science and Technology University, Addis, Ethiopia; ^3^Institutes of Geophysics, Space Science and Astronomy, Addis Ababa University, Addis, Ethiopia; ^4^Centers for Food Security Studies, College of Development Studies, Addis Ababa University, Addis, Ethiopia

## Abstract

In Ethiopia, more prevalent drought happenings have been documented in the past century. The problem has gradually expanded from the north to the rest parts with deepened intensity. The study aimed to examine the magnitudes of spatiotemporal patterns of drought at the Bilate watershed from 1981 to 2016. Monthly rainfall and temperature data were used for the analysis. The Standardized Evapotranspiration Indexes (SPEI) at SPEI-03 and SPEI-12 timescales were applied to evaluate the drought patterns. Among different drought indices, the SPEI is the most valuable and preferred Index for drought studies. The SPEI method considers the role of temperature than other indices to compare drought in time and space. The Mann–Kendall test was used for trend analysis. Accordingly, the result revealed that 1988–2016 were years of continuous drought events in both timescales with (SPEI = −2.5 to −1.2) drought value. Drought severity and frequency were highly detected at Wulberag areas (SPEI: −2.5). Durame, Angacha, and Alaba experienced increasing drought trends (*Z* = −1.96−1.6) and Welayita Sodo is *Z* = −0.07−0.03. Bilate-Tena and Hossana area of the watershed were less affected by drought than other areas. Spatially, the drought occurrences were observed in all areas of the watershed with varying magnitude. In the SPEI-12 timescale, more frequent drought occurrences were observed than SPEI-03. It was found that severe drought was observed in 1987, 1993/94, 2000–2005, and 2010. Moreover, the watershed experienced an Aridity Index (AI) of 0.43 (43%) and was subjected to potential high evapotranspiration (PET). The highest PET was observed at Bilate-Tena, Angacha, Hosanna, Wulberag, Alaba, Welayita Sodo, and Durame (151.6, 119.6, 119.3, 140.8, 142, 127.5, and 125.7 mm/year, respectively. Hence, the finding of this study could initiate a further inquiry on drought risk management, early warning responses, and local scale planning.

## 1. Introduction

Drought is among the foremost frequent hydro-meteorological hazards that affect the entire Earth's aspects. The occurrence of widespread drought across the Earth underlined the vulnerability of the environment and humanities to the adverse effect. The effect of drought is more comprehensive and multidimensional but felt slow [1–3]. Drought causes losses of life and impacts the livelihood of human beings [4]. Subsequently, it influenced the potentials of an ecosystem [5] and resulted in water stress, desertification, land degradation, and erosion [6]. Drought has been an exciting topic of research nowadays, mainly reconstructions of drought history, computations of drought frequency, and its impacts [7]. The occurrence of drought is not area-specific; it can occur in areas with high or low rainfall [1]. Drought is some long-term average condition of the imbalance between precipitation and evapotranspiration.

Drought might be classified as meteorological, hydrological, agricultural, and socioeconomic drought [7]. Usually, other droughts are preceded by the meteorological drought, i.e., deficiency of precipitation in a short duration [8]. Drought might be easily translated into disaster in developing countries, mainly which depends on rainfed agriculture. Several previous studies confirmed that the frequency and intensity of drought are increasing and are expected to continue increasing in the future [9]. As a result, the number of warm spells increased, whereas extremely cold days decreased and ended in the danger of drought for various world areas [10].

In Africa, particularly in Sub-Saharan countries, drought substantially impacts agriculture, human health, and the economy [9, 11, 12]. The recurrent drought across the Horn of Africa, including Ethiopia, is causing a widespread impact on water, agriculture, food security, and economy [10] and starvation [4]. In Ethiopia also drought has been identified as a hazard affecting the agriculture and water sectors, especially since the 1960s. Within the past century, quite 30 major drought episodes have occurred in Ethiopia, of which 13 covered the whole parts of the country [5]. Earlier studies highlighted that the magnitude and frequency of drought have increased in the country in time and frequency [11, 12]. The drought has devastated crops and killed many people and livestock [10, 12, 13]. In the years 1984,1985, and 2011, drought events affected many farmers and pastoralists in several parts of Ethiopia and affected the agricultural sector's contribution to the economy and food security [4, 5]. The distribution of the drought effect is not uniform across the country. The north and northwest regions of the country face frequent and more severe drought conditions than the southern and southwestern regions. A recent study confirmed that seasonal scale drought trends over the south and southwestern regions of the country during the spring season are frequently observed. Short-term drought was commonly observed in this particular season in the country's north, northwestern, and central mountainous regions [14]. Furthermore, the detected irregularity of drought over the various regions of Ethiopia is connected to sea surface temperature (SST) fluctuations [6].

A drought index is a number used to determine the magnitudes of drought events [15]. The drought indices measure the continuous precipitation deficit and temperature rise, decrease in river discharge, or another measurable variable [16]. Drought is quantified using various indices, such as the Palmer Drought Severity Index (PDSI), the Standardized Precipitation Index (SPI), and the Standardized Evapotranspiration Index (SPEI), which uses precipitation, temperature, and locally available water content based on the supply-demand concept of the water balance equation [17–19]. The recent SPEI, the most widely and commonly used drought monitoring index, investigated the drought events for this study. The SPEI is a multiscalar index, more suitable and expressive than other indices in terms of drought characterization, climate variability, and global warming [9, 12]. For instance, a study conducted in East Africa and northern Ethiopia [10, 14] proved that SPEI could be applicable in drought assessment in Ethiopia. Hence, this study evaluated the seasonal trend of the drought occurrence using the MK test with SPEI-03 and SPEI-12 timescales. The study helps to assess further drought, which is used to develop context-specific drought preventive measures.

## 2. Materials and Methods

### 2.1. Descriptions of the Study Area

**Figure 1:**
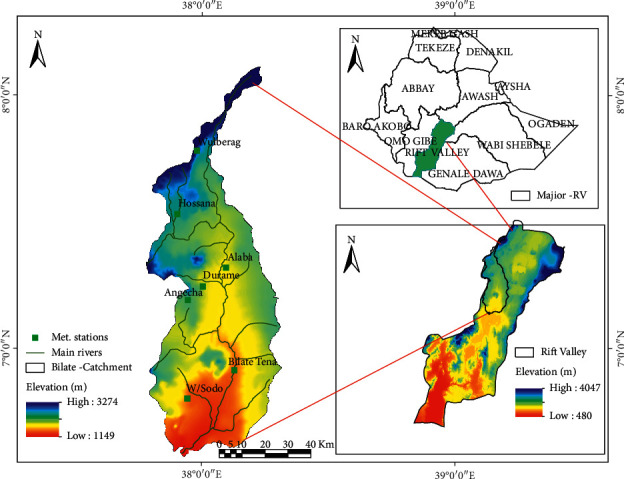
Map of the study area and meteorological stations.

The study site is located in the sub-basins of the Ethiopian Rift Valley in the southern region. It covers 5,330 km^2^ with varied topography ranging from plain to steep slope at the Mount Hambaricho (3028 m s l) in the Kembata area. The region has three distinct agro-ecological zones: tropical, subtropical, and temperate (Figure 1). According to FAO [20], eight types of soils are found in the watershed, which are suitable for the cultivations of maize, wheat, barley, teff, sorghum, enset, and vegetables. The spring (MAM) and summer (JJA) are the main cropping seasons in the watershed [21]. Enset is the most staple food for the community. The majority of communities in Kembata, Hadiya, and Welayita heavily depend on enset production and rearing of livestock as means of livelihood running.

### 2.2. Data Sources

Gridded data from 1981 to 2016 with a resolution of 4 × 4 km were obtained from the National Meteorology Agency of Ethiopia (NMAE). The high-resolution gridded data are essential for Ethiopia, where stations are scarce and sparsely distributed. The need to use gridded data is due to the incompleteness of observed and less spatial coverage [22].

### 2.3. Methods for Drought Analysis

Indices quantify drought at numerous spatiotemporal scales. Several drought indices have been identified to characterize the intensity and magnitude of drought based on user interest. For dependable drought hazard valuation and policymaking, a drought index that combines multiple variables should be used for more accurate drought monitoring [23]. This study characterized drought by duration, frequency, intensity, and trends. Rainfall data were further analyzed to investigate the changes in the frequency and extent of droughts conditions over the Bilate watershed. More than three decades of precipitation and temperature data were taken, and analysis was done. Since droughts are becoming a threat to most parts of the globe and commonly cover large areas and extend for long periods, studying such extreme climate events was essential. Droughts can be studied at any timescale using different indices (1, 3, 6,12, and 24 months), based on shorter (1, 3, 6) and longer timescales [17, 24]. SPI and SPEI are the robust indices most commonly used to compute drought conditions based on climatic data. SPEI is a widely used and recent index for drought analysis [14]. The values of the drought classes are indicated in Table 1.

#### 2.3.1. Standardized Precipitation Evapotranspiration Index (SPEI)

Standardized Precipitation Evapotranspiration Index(SPEI) [26] was used to assess drought conditions. It defines a drought index that is sensitive to climate variability and change. The precipitation deficit is the primary factor for the drought; air temperature, evapotranspiration, wind speed, and soil water holding capacity can also influence drought [14]. The SPEI is a multiscalar index, more suitable and expressive than other indices in terms of drought characterization, climate variability, and global warming [9,12]. The SPEI evaluates the difference between water supply and demand linked to temperature [27]. This work assesses drought situations by applying SPEI-03 and SPEI-12 in the Bilate watershed. The SPEI-03 is derived by averaging the 3-month values, i.e., Feb–April, May–July, Aug–Oct, and Nov–Jan, within a year, while SPEI-12 is from an accumulated 12-month timescale. A timescale of 3 months was chosen to denote drought impacts on agriculture during the four seasons. On the other hand, selecting a 12-month timescale aimed to reflect the hydrological consequences of drought [28]. Several methods are used to calculate the potential evapotranspiration (PET) [29]. The Hargreaves method was used to calculate PET in this study. The Hargreaves method desires only precipitation, minimum and maximum temperature, and latitude and is the extraterrestrial radiation (*R*_*a*_) [30].

(1) $$\begin{array}{c} PET_{HG}=0.0023\times \left(T_{mean}+17.8\right)\times \left(\sqrt{T_{\max}-T_{\min}}\right)\times Ra, \end{array}$$

where  *PET*_*HG*_ is the potential evapotranspiration of Hargreaves method (*mm*/*da*  *y*), *R*_*a*_ is the extraterrestrial radiation (mm/day), calculated as a function of latitude, *T*_mean_ is the average temperature ( C), and *T*_max_ and *T*_min_ are the maximum and minimum temperatures (*℃*). Once PET was estimated, *D*_*i*_  is calculated using the difference between precipitation and PET as in the following equation:

(2) $$\begin{array}{c} D_{i}=P_{i}-PET_{i}, \end{array}$$

where *D*_*i*_ is the climatic water balance (CWB) in a given period (*mm*), *P*_*i*_ is the precipitation in a given period (*mm*), and PET is the potential evapotranspiration in a given period (*mm*) calculated using the Hargraves method. Detailed computing of SPEI has been described in [14].

(3) $$\begin{array}{c} \text{SPEI}=W-\frac{C_{0}+C_{1}W+C_{2}W^{2}}{1-d_{1}W+d_{2}W^{2}+d_{2}W^{3}}, \end{array}$$

(4) $$\begin{array}{c} W=\sqrt{-2\;\ln\left(p\right)}\text{ For }p\leq 0.5, \end{array}$$

where *p* is the probability of exceeding a determined *D* value, *p* = 1-F (*x*). If *p* > 0.5, then *p* is replaced by 1-*p* and the sign of the resultant SPEI is reversed; the constants are *C*_0_ = 2.515517, *C*_1_ = 0.8022853, *C*_2_ = 0.010328, *d*_1_ = 1.432788, *d*_2_ = 0.189269, and *d*_3_ = 0.001308. Detailed computing of SPEI has been widely described in [14, 31].

#### 2.3.2. Trend Detection in Time Series of SPEI

In the current study, the drought trend has been done using the nonparametric Mann–Kendall test (MK) [6, 32]. The MK test is used to evaluate the statistical significance of increasing and decreasing trends of meteorological variables at the SPEI-3 and SPEI-12 timescales. The MK is a nonparametric statistical method and the most robust as well as suitable for detecting trends since it is less sensitive to outliers within time series data. The MK statistic (*S*) is mathematically computed as follows:

(5) $$\begin{array}{c} S=\overset{N-1}{\underset{i=1}{\sum }}\overset{N}{\underset{j=i+1}{\sum }}\text{sgn}\left(x_{j}-x_{i}\right), \end{array}$$

where *N* is the number of data points. Assuming (*x*_*j*_ − *x*_*i*_)=*θ*, the value of *sgn*(*θ*) is computed as follows:

(6) $$\begin{array}{c} \mathrm{sgn}\left(\theta \right)=\left\{\begin{array}{c} 1\;if\;\left(x_{j}-x_{i}\right)>0\\ 0\;if\;\left(x_{j}-x_{i}\right)=0\\ -1\;if\;\left(x_{j}-x_{i}\right)<0 \end{array}\right\}, \end{array}$$

where seasonal and annual values in years are *j* and *i*, *j* > *i*, respectively. (*x*_*j*_ − *x*_*i*_) is the sigma function. The test statistic (S) has been assumed to be asymptotically normal, *E*(*S*)=0. A positive value of S indicates an increasing trend, whereas a negative value indicates a declining trend in the data. For *n* ≥ 10, the statistic *S* is approximately normally distributed with the mean, and E(S) becomes 0 [33]. It is necessary to compute the probability associated with *S* and the sample size, *n*, to statistically quantify the significance of the trend. Statistical Kendall's *τ* (tau) test can be compared [34].

(7) $$\begin{array}{c} \tau =\frac{S}{N\left(N-1\right)/2}, \end{array}$$

where  *τ* has a range of −1 to +1 and is analogous to the correlation coefficient in regression analysis. The null hypothesis of no trend is rejected when *S* and *τ* are significantly different from zero. In this case, the variance statistic is given as

(8) $$\begin{array}{c} V\left(S\right)=\frac{1}{18}\left[n\left(n-1\right)\left(2n+5\right)-\overset{g}{\underset{k=1}{\sum }}t_{k}\left(t_{k}-1\right)\left(2t_{k}+5\right)\right], \end{array}$$

where *n* is the number of data points, *g* is the number of tied groups (a tied group is a set of data having the same value), and *t*_k_ is the number of data points in the *k*^th^ group. The standard test statistics *Z* is calculated as follows:

(9) $$\begin{array}{c} Z_{s}=\left\{\begin{array}{cc} \frac{S-1}{\sqrt{\text{Var}\left(S\right)}}, & S>0,\\ 0, & S=0,\\ \frac{S+1}{\sqrt{\text{Var}\left(\text{S}\right)}}, & S<0. \end{array}\right. \end{array}$$

The statistical significance level of the trend variation was evaluated using *Z*_*s*_ value. A positive *Z*_*s*_ value indicates an increasing trend while a negative *Z*_*s*_ shows a decreasing trend. Two-sided test under a significant *α* level, if |*Z*_*s*_| < *Z*_(1 − *α*/2)_, the hypothesis that the sequence *X*_*i*_ has no trend or *H*_o_ is accepted, but if |*Z*_*s*_| > *Z*_(1 − *α*/2)_,   the null hypothesis (Ho) is rejected, and the alternative hypothesis (H_1_) is accepted, then the sequence has either increasing or decreasing monotonic trend. *p* value is used to test the hypotheses: *H*_o_: no trend and *H*_a_: monotonic trend (upward or downward).

#### 2.3.3. Sen's Slope Estimator

Sen's estimator [35] has been widely used to detect the trend direction and determine the time series' magnitude [34, 36]. It is a nonparametric method that can calculate the change per unit time. This method assumes a linear trend in the time series; in this method, the slopes  *T*_*i*_ of all data pairs are calculated as follows [37]:

(10) $$\begin{array}{c} T_{i}=\frac{x_{j}-x_{i}}{j-i}. \end{array}$$

For *i*  = 1, 2, *N,* where *x*_*j*_ and *x*_*i* _ are data values at a time  *j* and *i* (*j* > *i*), respectively. If there are *n* values  *x*_*j*_  in the time series and obtained *N* = *n* (*n*−1)/2, slope estimates *S*_*i*_. The median of these *N* values of *T*_*i*_ is Sen's estimator of the slope, which is calculated as

(11) $$\begin{array}{c} T_{\text{Med}}=\left\{\begin{array}{cc} T_{N+1/2}, & N\text{ is odd,}\\ \frac{1}{2}\left(\frac{T_{N}}{2}+\frac{T_{N+2}}{2}\right), & N\text{ is even,} \end{array}\right. \end{array}$$

A positive value indicates an increasing trend, and a negative value indicates a decreasing trend in the time series. Finally, the median tested with a two-sided test at the 100 (1−*α*)% confidence in the true slope may be obtained with the nonparametric test [38]. In this study, the frequency of drought incidences was evaluated by considering the number of SPEI values ≤ −1,

(12) $$\begin{array}{c} F=\frac{n}{N}*100, \end{array}$$

where *n* is the number of months' drought events (SPEI ≤ −1) that an index value meets a set of drought criteria divided by the number of months in the entire series (N). Drought frequency (F) was used to assess the drought prevalence during the study period [39].

To further analyze the spatial variation of drought, inverse distance weight (IDW) method was used to interpolate the probability of various drought events [40].

### 2.4. Aridity Index

Aridity is associated with drylands, sparse vegetation, sand dunes, small amounts of water, no surface water, and high temperatures described as a desert-like condition. This is not always accurate as aridity is a climate phenomenon principally characterized by a water shortage. Thus, aridity also occurs in cold climates where precipitation falls mainly due to snow. Potential evapotranspiration (PET) measures the “drying power” of the atmosphere to get rid of water from land surfaces by evaporation via transpiration. Consequently, the climate is considered arid if PET is more significant than P [41].

The Aridity Index (AI) is a convenient but straightforward numerical indicator of aridity supported long-term climatic water deficits and is calculated because of the ratio P/PET. AI is a numerical indicator of the degree of dryness at a given location. The AI of an area can be expressed in terms of rainfall and evapotranspiration [42, 43]. Several aridity indices have been estimated, which helps identify areas that suffer from a moisture deficiency. This situation can severely disturb the actual usage of the land for cultivation. The Aridity Index is helpful to determine the extent of drought severity. Numerous aridity indexes were established, mainly relating to PET [44, 45]. Using the AI, various types of drylands are classified as indicated in Table 2. An Aridity Index (AI) can be computed as

(13) $$\begin{array}{c} \text{AI}=\frac{\text{P}}{\text{PET}}. \end{array}$$

### 2.5. Conceptual Framework for Drought Analysis

**Figure 2:**
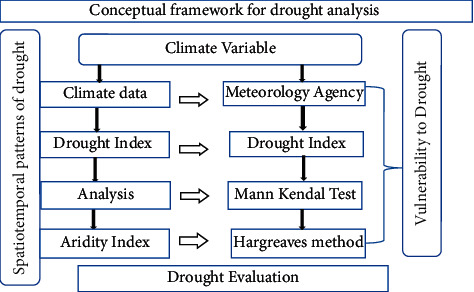
The conceptual framework illustrating the spatiotemporal patterns of drought.

The overall structure of the study is demonstrated in the conceptual framework that incorporates all necessary procedures followed in the study (Figure 2).

## 3. Result

### 3.1. Temporal Patterns of Dry/Wet Incidences

**Figure 3:**
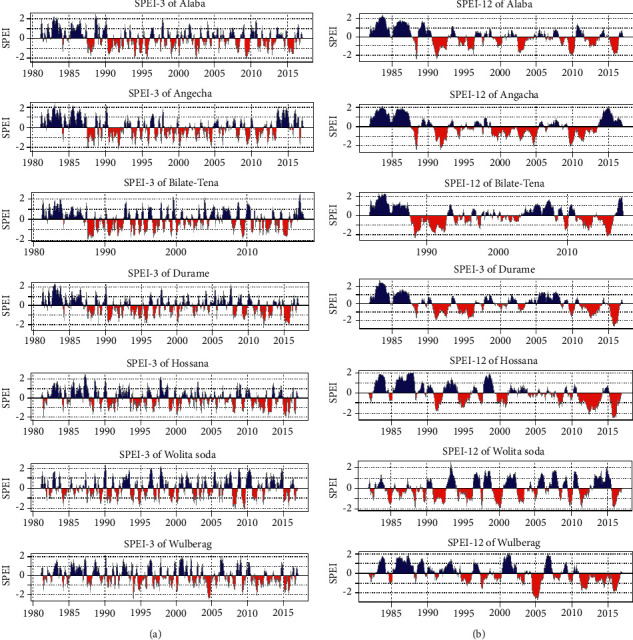
Mean SPEI-3 (a) and SPEI-12 (b) over Bilate watershed showing the variation in the duration, severity, and intensity of dry and wet events, 1981–2016.

Figure 3 illustrates the temporal patterns of drought conditions at SPEI-03 Figure 3(a) and SPEI-12 Figure 3(b) in Bilate watershed for 1981–2016. Dry and wet events were evaluated as moderate, severe, and extreme based on the classification standard provided in Table 1. The SPEI-03 results in Figure 3(a) indicated the drought conditions at SPEI-03, which verified that the watershed experienced moderate, severe, and highly severe drought in the past 36 years. The downward strip (below zero) indicates the occurrence of drought with a varying magnitude; as the strip gets downward negative, the drought magnitude increases and vice versa.

The severe drought extremes observed in 1987, 1991, 1995, 1996, 2000, 2003, and 2004 are marked as arid years with the SPEI value ranging between −2.5 and −1.2 as per drought severity class, whereas wet extremes were observed in 1983, 1986, 1990, 1996, 2001, 2004 2010, and 2015/16. Stationwise, extreme wetness was observed in Durame, Hossana Bilate-Tena, Angacha, Welayita Sodo, and Hosanna in the years 1983,1987,1988,1990, and 1997, respectively. The drought years identified in the study periods correspond to sea surface temperature (SST), which affects the seasonal precipitation patterns in the watershed. Concerning SPEI-12 (Figure 3(b)) indicates extreme dryness condition in 1988 and 1991–1993 with the value of −2.5 depicting severe drought, except Wulberag station while 1995/96 moderate drought was observed at all stations. At stations, Angacha, Wulberag, Alaba, Durame, Hossana, and Bilate-Tena, in the years 1999–2006, 2005, 2010, and 2015/16, extreme drought events were detected, respectively, with an extreme severity classification of −2.5, whereas the rest of the stations experienced wettest extremes relatively as illustrated in Figure 3.

Table 3 portrays the frequency of dry and wet events at the Bilate watershed in the study periods. The frequency of dryness events for SPEI-03 ranges from 12 to 18. The highest frequency of drought events was observed at Welayita Sodo station, whereas the lowest frequency of drought was detected at Angacha station for SPEI-03. In this timescale, the wetness frequency ranges from 11 to 20. The frequency of dryness events for SPEI-12 ranges from 7 to 12; the maximum frequency of dryness was observed at Wolayita Sodo.

In contrast, less frequency of dryness was observed at Wulberag station. The result obtained indicates that the frequency of wetness and dryness for SPEI-03 is more than SPEI-12. The results for the SPEI-12 timescale showed stability in the frequency of incidences of drought. This proves that SPEI at elongated (SPEI-12) timescales responded gradually and consistently to deviations in climatic variables, indicating intense durations of frequent occurrences of anomalous events over the years.

### 3.2. The Spatial Variation of Drought at the Watershed

**Figure 4:**
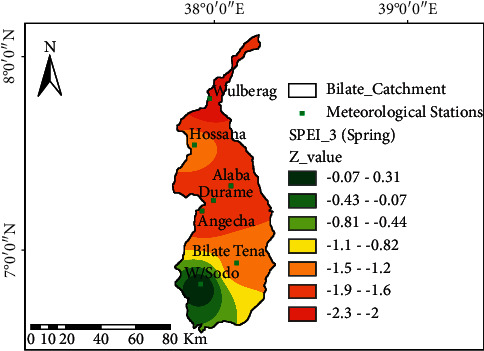
Spatial characteristics of drought in spring using SPEI-03; the black dot at the spatial map shows the location of meteorological stations.

**Figure 5:**
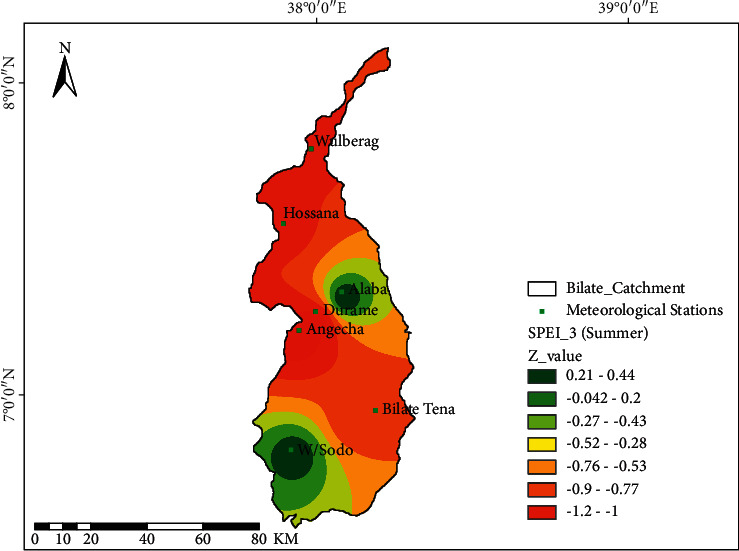
Spatial characteristics of drought in summer using SPEI-03; the black dot at the spatial map shows the location of meteorological stations.

**Figure 6:**
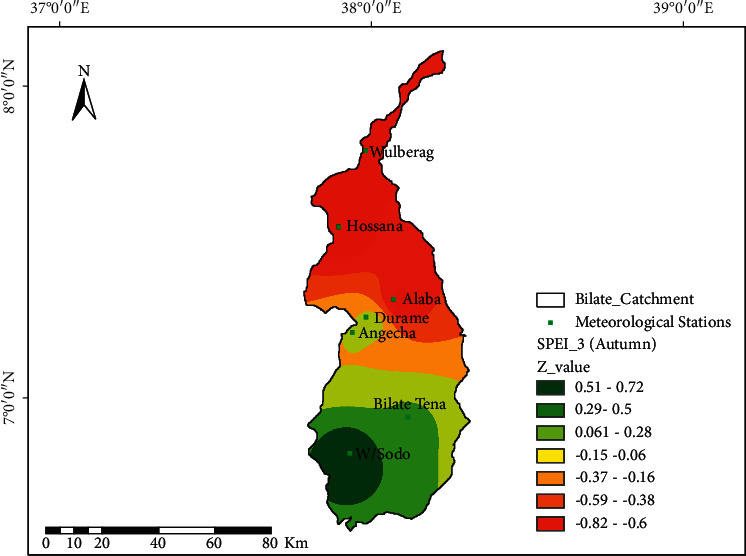
Spatial characteristics of drought in autumn using SPEI-03; the black dot at the spatial map shows the location of meteorological stations.

**Figure 7:**
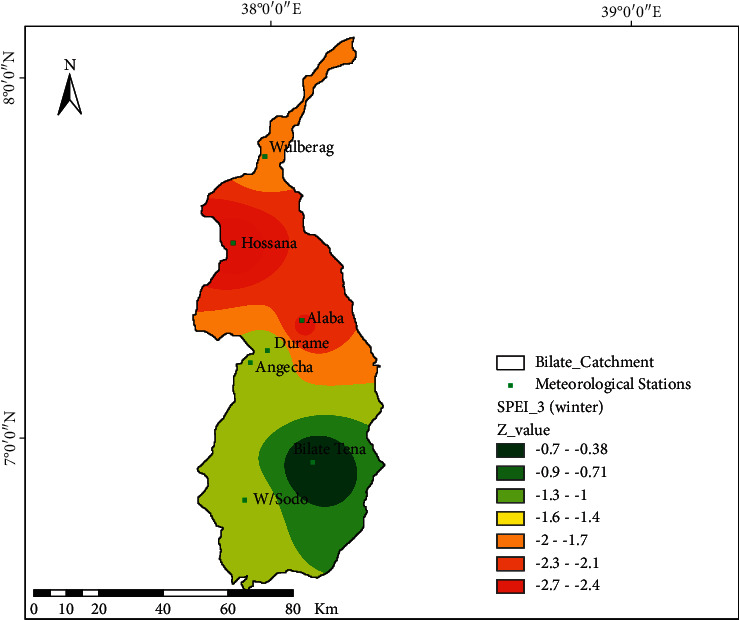
Spatial characteristics of drought in winter using SPEI-03; the dot at the spatial map shows the location of meteorological stations.

**Figure 8:**
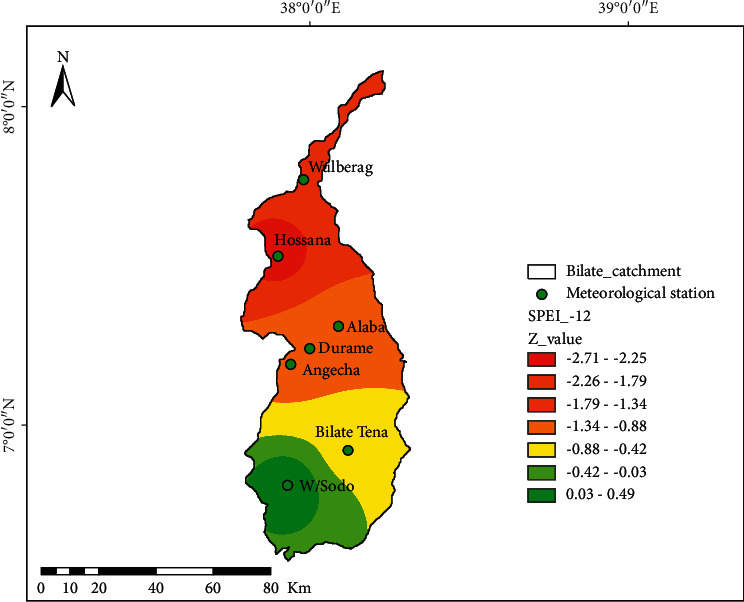
Spatial characteristics of drought at SPEI-12; the black dot at the spatial map shows the location of meteorological stations.

The spatial patterns of SPEI-03 for spring, summer (JJA), autumn (SON), and winter (DJF) seasons are illustrated in Figures 4–7 and the SPEI-12 in Figure 8. The Z-value is employed to demonstrate the drought trend at the watershed based on severity class. The negative value of “Z” indicates the presence of drought of various classes, and the positive value indicates wetness. The result in Figure 4 described that Welayita Sodo areas of the watershed showed a less dryness trend (*Z* = −0.07−0.03), and Wulberag areas of the watershed were highly hit by severe drought having significant trends of *Z* = −2.5 in spring. Durame, Angacha, and Alaba experienced similar drought conditions (*Z* = −1.96−1.6). Bilate-Tena and Hossana area experiences less dryness of spring drought than other areas in the watershed. The drought conditions increase from the south to the north of the watershed. During the spring season, the Welayita Sodo area is in a moderate wet situation that increases towards the north and Wulberag is in extremely dry condition (Figure 4).

Figure 5 demonstrates the spatial characteristics of drought at the Bilate watershed during the summer (JJA) season. The summer season locally known as Kiremt is the primary rainy season at the watershed. The bulk of the watershed areas relies on summer precipitation for agricultural production and livelihoods. The scarcity of summer rainfall highly affects the livelihood of the communities residing at the watershed. The SPEI findings show that W/Sodo and Alaba areas less affected by drought conditions in summer seasons show insignificant trends of *Z* = 0.21–0.44 and −0.042–0.2) with varying degrees of dryness. The western margin of the watershed, which encompasses the area of Angacha, Durame, Hossana, and Wulberag with the trend values of *Z* = −1.2 to −1 and Bilate-Tena area (−0.76 to −0.53), was highly affected by drought conditions with a varying extent of dryness (Figure 5).

Figure 6 depicts the spatial distributions of drought characteristics at Bilate watershed during the autumn (SON) season, which is locally known as the “Tseday” season. It is noted that Welayita Sodo and Alaba areas of the watershed were less affected by the autumn/Tseday/drought. The SPEI-03 indicates insignificant positive trends of *Z* = 0.21–0.44 for both stations. The northern half of the watershed (, Hossana, and Alaba) is hit by the drought of moderate class (*Z* = −0.8 to −0.6); Durame and Angacha at the central parts of the watershed are not affected by drought during this season (*Z* = 0.061–0.28); Welayita Sodo and Bilate-Tena receive better distributions of moisture/wetness (*Z* = 0.51–0.72). In autumn, Welayita Sodo areas experiencing moderately wet conditions and moderately dry conditions at Wulberag was detected (Figure 6).

Figure 7 demonstrates the spatial distributional characteristics of drought at the Bilate watershed during the winter season (DJF). The winter season is usually known for its dryness, and no more precipitation is expected at the watershed. The Z-value in the spatial plot proves that Wulberag, Durame, and Angacha districts/stations are affected by severe drought trends, (*Z* = 1.6 to 1.3) conditions, whereas Bilate-Tena and its surrounding area capture better distributions of moisture than other areas. Apart from only a few pocket areas during the winter season, almost no precipitation is observed in parts of the Bilate watershed altogether; however, moderately dry condition at Bilate-Tena, severe drought at Wulberag, and extreme drought at Hosanna were noticed (Figure 7).

The drought characteristics at the longer duration (timescale), i.e., SPEI-12, are shown in spatial Figure 8: it had been found that in the Hosanna area, extreme dryness was observed with the significant value of *Z* = −2.71 to −2.25 indicating extremely dry circumstances at Hosanna regions of the watershed. The second worst drought class was observed at one among the districts named Wulberag with the SPEI magnitude of *Z* = −2.25 to −1.79, indicating extremely dry conditions. One can say the drought condition was highly pronounced at Wulberag areas of the watershed. The rest of the areas such as Angacha, Durame, and Alaba fall into an equivalent drought severity class (*Z* = −1.79 to −1.34). Bilate-Tena at the southern parts of the watershed had experienced moderately drier conditions (*Z* = −1.34 to −0.88). The southwestern parts of the watershed capture better wetness extreme than other areas. For instance, the Welayita Sodo region had experienced wetness of +0.03 to -0.49 at the SPEI-12 timescale. Generally, the drought severer drought class is observed in SPEI-12 compared to SPEI-03 at the entire Bilate watershed (Figure 8).

### 3.3. Drought Trend Analysis Using SPEI

The MK test analysis indicated an insignificant drought trend at Alaba, Hosanna, Bilate-Tena, and Wulberag for SPEI-03. However, an insignificant wetness trend was observed for SPEI-03 in Angacha, Durame, and W/Sodo stations. For SPEI-12, in almost all of the watershed, a significant drought trend was observed. The result revealed that the magnitude of drought at SPEI-12 was more frequent and intense than SPEI-03 (Table 4).

### 3.4. Potential Evapotranspiration

Table 5 demonstrates the potential evapotranspiration for each station at Bilate watershed. Among the stations, Bilate-Tena has high potential evapotranspiration (151.6 mm/year) than Angacha and Hosanna which have the lowest potential evapotranspiration 119.6 and 119.3 mm/year, respectively. The rest of the stations Wulberag (140.8), Alaba (142), Welayita Sodo (127.5 mm/year), and Durame (125.7 mm/year) stations' relatively similar potential evapotranspiration was observed. The average potential evapotranspiration observed at the watershed is 115.41 mm/year.

### 3.5. Aridity Index (AI)

An Aridity Index (AI) is a numerical indicator of the degree of dryness of the climate at a given location (Table 2). These indicators serve to identify, locate, or delimit regions that suffer from a deficit of available water, which can severely affect the actual use of the land for activities like agriculture or stock-farming. It was found that the watershed ranges between arid, semiarid, and dry subhumid in terms of aridity. The districts Hosanna, Angacha, and Welayita Sodo were less affected by aridity problems, whereas Alaba, Bilate-Tena, and Wulberag were the districts with high incidences of drought (Table 6). The average Aridity Index of the watershed was found to be 0.05 for the last three decades of time.

## 4. Discussion

Both precipitation and temperature disparities directly contribute to drought from moderate to severe drought. Drought happens when precipitation deviates from its average for consecutive months, mainly its effect was boldly seen during the cropping season [47]. To determine the possible risk of droughts happening in the future, it is vital to thoroughly analyze the historical drought events. The impact of drought depends on severity, duration, and spatial extent [48]. In Ethiopia, rainfall has become more erratic, and the frequency of drought has been increased [49,50]. Usually, drought occurs once every 7 to 10 years, but nowadays, it occurs every 3 to 4 years and the frequency of the drought occurrence is highly frequent [51–53]. The watershed is divided into three agro-ecological zones (highland, middle, and low land) to simplify drought characteristics. From highland agro-ecology, Angacha and Durame in the Kembata area and Hossana from Hadiya areas were commonly hit by moderate drought (Figure 1). The middle agro-ecology encompassing Wulberag (silite), Alaba, and parts of Bilate-Tena stations from Welayita Sodo regions experiences severe drought conditions. Generally, all forms of drought in the study area were observed frequently. The findings of this study were in agreement with the conclusions of [49,54,55].

SPEI Index indicates that all negative values (<−2) mean the watershed experienced extreme drought conditions in 1987–1993, 200–2005, and 2010–2012 both spatially and temporally. However, in the case of lowland areas of the watershed, drought condition is more severe than the higher and middle agro-ecology. Bilate-Tena station experienced high temperatures throughout the study period and was vulnerable to severe drought. The result depicted that this station is frequently affected by drought calamities compared to others. The analysis of SPEI revealed that drought occurred at a different level of severity from 1981 to 2016, particularly in the main cropping season, and complicated the livelihoods of the communities dwelling at Bilate watershed. The study's findings also showed that the years 1987 to 1995 were years of continuous severe and frequent drought. This indicates low rainfall distribution was so pitiable, and the high temperature was observed at all drought analysis timescales; therefore, this part of the watershed is more susceptible to both extremely severe and severe drought incidents as compared to the rest of the station; these findings are supported with the earlier study done by [56].

The values of SPEI-03 and SPEI-12 confirmed that 2002–20003 were the major drought years and threatened the fundamental aspects of life. A similar study reported that over 10 million people required food support due to drought in Ethiopia [57]. The overall scenario signals that the country faces a complex problem associated with drought incidents [57–59]. To monitor the occurrence of drought, one should know the details and characteristics of spatial patterns of precipitation and its magnitude. SPEI is one of the most important indices to evaluate wetness or dryness and is mainly used for countries like Ethiopia [57]. In this investigation, both short-term and long-term SPEI values confirmed that the drought category fell from severe to extremely severe. Both the conditions are hazardous for agriculture productivity and entirely affect the livelihoods of farming communities and their stock [60].

As the study witnessed that frequency of dry and wet event occurrence for SPEI-03 was greater than SPEI-12, however, the duration of SPEI-12 is longer than SPEI-03. The results for SPEI-12 showed stability in the incidences of drought events. This demonstrates that the SPEI at long-term timescales (SPEI-12) responded gradually to deviations in climatic variables. Then, the longer timescales were most appropriate for revealing the incidences of drought over the region, whereas shorter intervals demonstrated suitability for detecting frequent seasonal and interannual variations [61,62].

MK trend analysis revealed that drought trends have increased in space and time across Ethiopia. These results showed an overall increasing trend of drought throughout the study periods. These differences in trends were likely due to changes in climatic and environmental conditions in the past three decades. In addition, the global sea surface temperature fluctuations and seasonal and rainfall characteristics might have also contributed to the differences in timescale and frequencies of drought. The increasing drought trends indicated that the number of drought years increased at the Bilate watershed, thus causing damage to agriculture and water resource facilities. This study is in agreement with the findings mentioned in earlier studies [63,64]. The negative SPEI values for all timescales suggested that drying events might have been amplified over the watershed. The most severe drought years were 1987–93, 2000–2005, and 2010–2019, which resulted in larger impacts on the environment and society [6,32]. The previous study confirmed that drought severity is location and livelihood specific and determined by its frequency and magnitude [65,66].

Spatially, increasing drought trends were more at five of the stations; the remaining two of the stations found in the watershed are less affected by droughts events. The findings of this study are associated with the observations of [57,67]. This can lead to prolonged drought over the areas during scarce or low precipitation at the watershed. Another critical point of this investigation was the potential evapotranspiration (PET) and the aridity characteristics. PET is a measure of the amount of water that would be evaporated and transpired if sufficient water was available. The findings confirmed that the highest PET was observed at Bilate-Tena, Angacha, Hosanna, Wulberag, Alaba, Welayita Sodo, and Durame with the value of 151.6, 119.6, 119.3, 140.8, 142, 127.5, and 125.7 mm/year, respectively. In terms of dryness, due to fluctuating weather conditions, the study area experiencing the highest aridity conditions in the study periods with aridity value of 0.43 (43%) indicated an adverse impact on the agrarian community.

### 4.1. Drought Hazards and Future Concerns

Knowing the historical characteristics of drought occasions is very crucial for policymaking and proposes a coping mechanism and early warning to minimize the disasters caused by drought. Identifying the magnitudes and causes of drought helps design the forthcoming mitigation options using the past as a base. The findings of this particular study and other similar studies confirmed that the frequency, intensity, and magnitudes of drought are sharply increasing from time to time in Ethiopia and seriously challenging rainfed agriculture. Drought affects all parts of the country with varying magnitude, mainly attributed to natural climate variations and change. In Ethiopia, climate variability can trigger the occurrence of drought [67, 68]. The rising drought pattern influences man's activities, particularly the agrarian community and easy functioning of the ecosystem, and hinders sustainability. Some research has recommended that geographic areas at the horn of Africa, including Ethiopia, would be affected by extensive droughts in the forthcoming century [69]. The drought situations might be intensified due to man-made effects, climate variability/change, and environmental degradations. The study suggested that the snowballing dryness trends would exacerbate global warming [70–72]. Rising global temperature makes geographic areas more susceptible to drought catastrophes [14]. Increasing land degradation, unwise utilization of natural resources, and climate change accelerate drought occurrences [73, 74]. To minimize the impacts caused by drought, there must be an inclusive approach to adaptation and mitigation priorities to control the adverse effect of dryness in the coming centuries. The communities and government should think of which helps lessen the impacts of drought in Ethiopia in general and Bilate watershed in particular.

## 5. Conclusion

The study emphasizes describing the magnitude of drought in the Bilate watershed at the sub-basins of the Ethiopian Rift Valley. The study used the SPEI to analyze the spatiotemporal patterns of drought from 1981 to 2016. The drought conditions (duration, frequency, and intensity) at short- and long-term timescales were investigated (SPEI-03 and SPEI-12). Among the various drought indices, Standard Evapotranspiration Index (SPEI) had been chosen for this study due to its advantages over other drought indices. The SPEI considers the role of temperature through its influence on potential evapotranspiration. The SPEI may be widely utilized in assessing the impact of worldwide warming on droughts. The SPEI is a relatively new drought index, comprises a temperature component, and takes into account the effect of temperature on drought development through a basic water balance calculation. The SPEI may be a power scale during which both positive and negative values are calculated; it identifies wet and dry events at a look. It is multiscalar and often calculates for one month to 48 months or more. The SPEI is acceptable for all climate regimes. It is a perfect index for assessing climate impact studies and modeling various climate scenarios. However, the SPEI requires a complete dataset for temperature and precipitation, limiting its use due to insufficient data being available. Rapidly developing drought situations might not be identified quickly as a monthly index.

Overall, an increasing drought trend was found in the study period with distinct spatial and temporal drought patterns. The drought conditions have increased from time to time. 1987 onwards almost all the months were not free from the impact of drought. 1987 was taken as a turning point for the occurrence of drought with high fluctuation with substantial regional variations across the varied agro-ecology in the watershed. The study area has experienced the most severe droughts during 1987–1993, 2000–2005, and 2010–2012, which led to serious damage to societies and the environment. The prolonged durations of drought events are inclined to increase with the SPEI timescale. The result of SPEI at all timescales displayed negative trends, thus designating that drought has increased in the past three decades. The MK trend test and slope indicated that the long- and short-term drought condition has increased. The findings indicated that the studies have important implications for creating drought management strategies and adaptation measures.

## Figures and Tables

**Table 1:** Categorization of drought and its equivalent index, SPEI [25].

SP0EI	Extremely wet	Severely Wet	Moderately wet	Near normal	Moderately dry	Severely dry	Extremely dry
	≥2.0	1.50–1.99	1.49–1	0.99–0.99	−1.00 to −1.49	−1.5–1.99	≤−2.00

**Table 2:** Aridity classifications [46].

Aridity Index	Hyperarid	Arid	Semiarid	Dry subhumid
AI	<0.05	0.05 < AI < 0.20	0.20 < AI < 0.50	3.50 AI < 0.65

**Table 3:** Frequency of wet and dry events of SPEI-03 and SPEI-12.

Event	SPEI	Frequency of severe wet and dry events						
		Alaba	Angacha	Durame	Hossana	Wulberag	W/Sodo	Bilate-Tena
Wet	03	15	11	12	15	15	20	18
	12	4	3	5	5	7	11	7

Dry	03	13	12	17	16	18	18	15
	12	7	9	8	7	6	15	7

**Table 4:** Trend analysis of Standardized Evapotranspiration Index.

Index	Angacha	Alaba	Durame	Hosanna	Bilate-Tena	Wulberag	W/Sodo
SPEI-03	0.07	−0.67	0.17	−0.82	−0.33	−0.74	0.72
SPEI-12	−1.43	−1.56	−1.9	−2.71	−1.10	−2.18	0.49

**Table 5:** Potential evapotranspiration from the station results at Bilate River watershed.

	Wulberag	Alaba	Angacha	Hosanna	Durame	W/Sodo	Bilate-Tena
Mean	140.8	142.96	119.6	119.3	125.7	127.5	151.6

**Table 6:** Aridity Index of districts at Bilate watershed from 1981–2016.

Station	Angacha	Durame	Hosanna	Wulberag	W/Sodo	Bilate-Tena	Alaba
AI	0.69	0.64	0.74	0.60	0.76	0.25	0.19

## Data Availability

The data used to support the findings of this study are available from the corresponding author upon request.
